# Churn prediction in telecommunication industry using kernel Support Vector Machines

**DOI:** 10.1371/journal.pone.0267935

**Published:** 2022-05-24

**Authors:** Nguyen Nhu Y., Tran Van Ly, Dao Vu Truong Son

**Affiliations:** School of Industrial Engineering and Management, International University, Vietnam National University, Ho Chi Minh City, Vietnam; Universiti Malaysia Pahang, MALAYSIA

## Abstract

In this age of fierce competitions, customer retention is one of the most important tasks for many companies. Many previous works proposed models to predict customer churn based on various machine learning techniques. In this study, we proposed an advanced churn prediction model using kernel Support Vector Machines (SVM) algorithm for a telecom company. Baseline SVM models were initially built to find out the most suitable kernel types and will be used to make comparison with other approaches. Dimension reduction strategies such as Sequential Forward Selection (SFS) and Sequential Backward Selection (SBS) were applied to the dataset to find out the most important features. Furthermore, resampling techniques to deal with imbalanced data such as Synthetic Minority Oversampling Technique Tomek Link (SMOTE Tomek) and Synthetic Minority Oversampling Technique ENN (SMOTE ENN) were used on the dataset. Using the above-mentioned techniques, we have obtained better results compared to those obtained from previous works, we achieved an F1-score and accuracy of 99% and 98.9% respectively.

## Introduction

Telecommunications is known as one of the fast-growing industries in many countries. However, the average annual churn rate of the telecom industry is between 20–40% [1], which leads to huge loss of revenue. Customers always have a variety of choices, and they tend to choose the companies that can offer them better quality and less expensive services. To survive in this highly competitive market, telecom companies have to develop strategies in attracting new customers or increasing the customer retention rate. It is noted that acquiring a new user costs 5 to 10 times more than retaining an existing one [1]. Therefore, customer churn prediction has become a popular research area since it has the potential to help telecom companies to identify customers with high potential of terminating their contracts. Then, companies evaluate the situations and design appropriate service packages for these customers to retain them. It is extremely important for the company’s long-term development.

Many previous works [1–10] have confirmed that customer churn prediction depended on complex human behaviors, such as customer data, usage, consumption behavior, and so on.

Typical a churn prediction model involves four steps: (1) data gathering and understanding; (2) features selection; (3) design and development of predictive model; (4) validate and evaluate the model.

Most previous studies focused on improvements of predictive models using machine learning algorithms [1–10] such as neural networks, support vector machine, random forest, etc. However, prediction results are not highly accurate due to incorrect classifications of churners and non-churners.

In this work, we proposed an integrated framework for churn prediction problem using: (1) a data augmentation technique to improve class imbalance in the dataset; (2) a feature subset selection using Sequential Forward Selection (SFS) and Sequential Backward Selection (SBS); (3) a kernel support vector machine as the predictive model.

Our paper is organized as follows. Section 2 discusses related work. Section 3 discusses the proposed methodology, including: (1) material/method; (2) data collection/input and preprocessing; (3) feature selection; (4) our support vector machine. Section 4 describes result analysis; and Section 5 presents the conclusion and future work of the paper.

## Related works

### Customer churn

**Fig 1:**
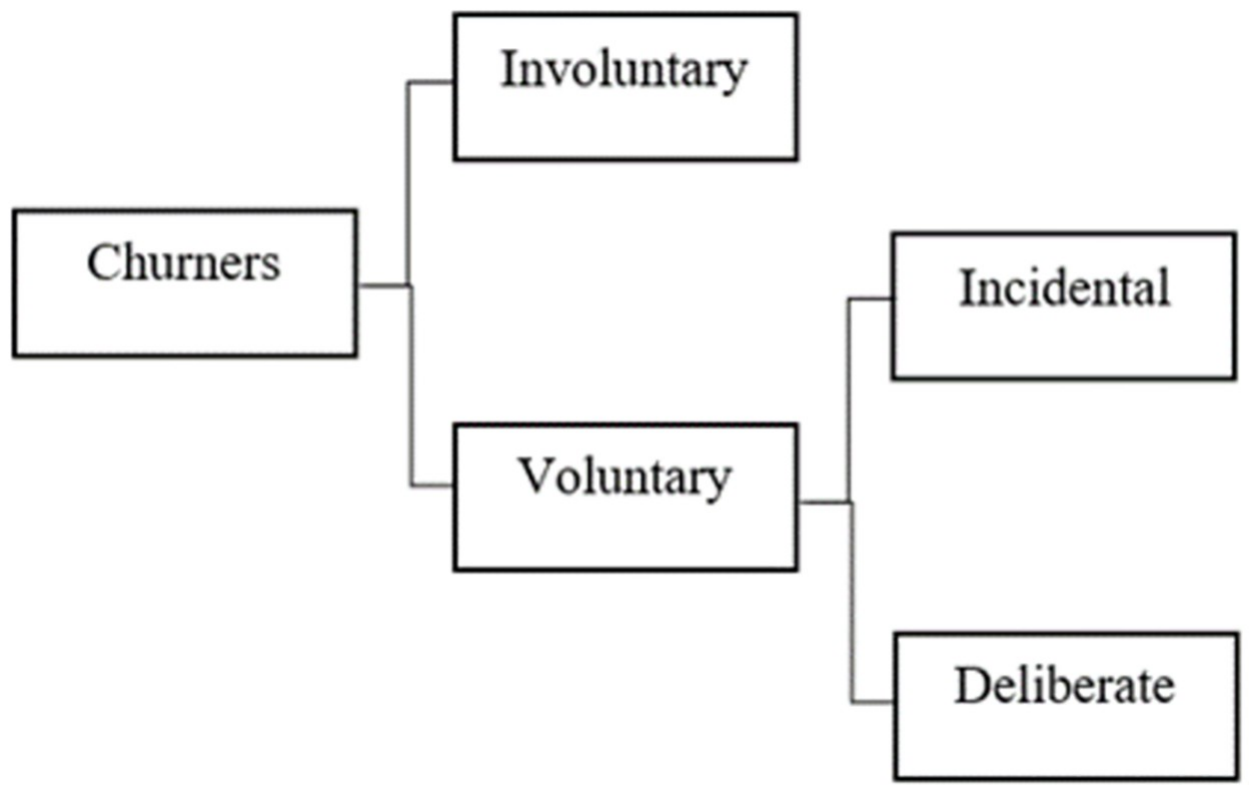
Types of churners.

It can be understood that customer churn in telecom industries are the ones who start switching from one service provider to another. As can be seen in Fig 1, churners can be classified into two main categories which are voluntary and involuntary [2].

It is easy to recognize churners who are involuntary. They are customers who cheat, have problems with payment or do not use the phone. Companies eliminate them from subscriber list proactively and have the ability of controlling and managing the risks of this churn kind.

In contrast, cases of voluntary occur when the customers actively come to a decision to stop using the service provided by a telecommunication company. It is difficult to detect this type of churn as well as determine the reasons why this class stopped using services. Thus, voluntary churners become the subject of most studies within this field [2]. They can be classified into sub-categories including incidental churn and deliberate churn. Incidental churners terminate the service contract since there are some changes occurring in their life. They may be jobless and forced to cease the service or they may move to a new location where the services are not available. In fact, this churn type accounts for account for only a small percentage of churn taxonomy. Most cases of churn happen when customers deliberately switch to use services of another competitor. They may find out or be offered services with newer technology, lower price, better quality. Their selection may also be affected by friends, family, self-image, and experimentation.

### Churn prediction

There were many models of churn prediction with the applications of several binary classification techniques. Table 1 is the summary of literature review.

**Table 1:** Literature review summary.

References	Dataset	The best classifier	Accuracy
Zhao et al., 2005 [3]	100000 instances, 171 attributes	Gaussian kernel Support Vector Machines	87.15%
Xia et al., 2008 [4]	3333 instances, 21 features	Radial basis kernel Support Vector Machines	90.88%
Shaaban et al., 2012 [5]	5000 instances, 23 attributes	Support Vector Machines	87.3%
Brandusoiu et al., 2013 [6]	3333 instances, 21 attributes	Polynomial kernel Support Vector Machines	85.63%
Rodan et al., 2014 [7]	5000 instances, 11 features	Radial basis kernel Support Vector Machines	94.3%
Keramati et al., 2014 [8]	3150 instances, 12 features	Hybrid methodology	95%
Zhou et al., 2019 [9]	3333 instances, 21 features	Polynomial kernel Support Vector Machines	91.67%
Ebrah et al., 2019 [10]	Dataset 1: 7033 instances, 21 features Dataset 2: 71,047 observations and 57 attributes	Support Vector Machines	Dataset 1: 87% Dataset 2: 99%
Kriti, 2019 [11]	7055 instances, 20 features	XGBoost	85%
Ullah et al., 2019 [12]	Dataset 1: 64,107 instances, 29 features Dataset 2: 3333 instances, 16 features	Random forest	Data set 1: 88.63% Dataset 2: 89.59%
Xin Hu et al., 2020 [13]	2681 instances	Hybrid methodology	98.87%
Panjasuchat et al., 2020 [14]	100000 instances, 99 attributes	Deep Q-Network	65.26%
Jain et al., 2020 [15]	3333 instances, 20 attributes	Logistic regression	85.24%
Mallika et al., (2020) [16]	7043 instances, 21 attributes	LightGBM	78.9%

Among several machine learning algorithms, Support Vector Machine has shown its outstanding performance in many previous studies. There are some previous studies using the same dataset as in [4, 6, 9, 15]. It can be seen from the results that kernel Support Vector Machines always outperformed others for churn prediction task, especially the model using Polynomial kernel in the research of Zhou et al. [9] which reached the highest accuracy of 91.67%. Jain et al. [15] proposed two approaches including Logistic Regression and Logit Boost but the accuracy generated from these methods were lowers than models using kernel tricks introduced in preceding studies which used the same dataset. Furthermore, in [3, 5, 7, 10], authors showed that kernel Support Vectors Machines gave the best results compared to other techniques. Each real-world dataset has its own properties and kernel tricks are very functional in constructing the type of space and hyperplanes of Support Vector Machine that are suitable with the studied dataset.

Among all the articles reviewed, accuracy was used as the most important metrics to evaluate the results. However, in most of cases, the datasets are highly unbalanced so that accuracy is not a good metric to assess the result and other metrics should be considered, except the case that balancing techniques were applied before classification step. Oversampling methods were used in [6, 9] to make population of two classes in the training set balanced. Another work using Synthetic Minority Oversampling Technique (SMOTE) proposed by Mallika et al. [16] resulted in an increased performances of classifiers from 3 to 5%. The results were not improved significantly since the techniques were only applied for training data and the testing sets were still imbalanced.

Another approach is to use a dimensionality reduction such as feature selection, before using classifiers. Better sets of features were selected by this approach, therefore, leads to an increased prediction efficiency. For instances, in [6, 10, 12], highly correlated features are removed from the original datasets. Mallika et al. [16] proposed models using Recursive Feature Elimination (RFE), which is a wrapper feature selection method to point out the best performing features.

## Material and methods

**Fig 2:**
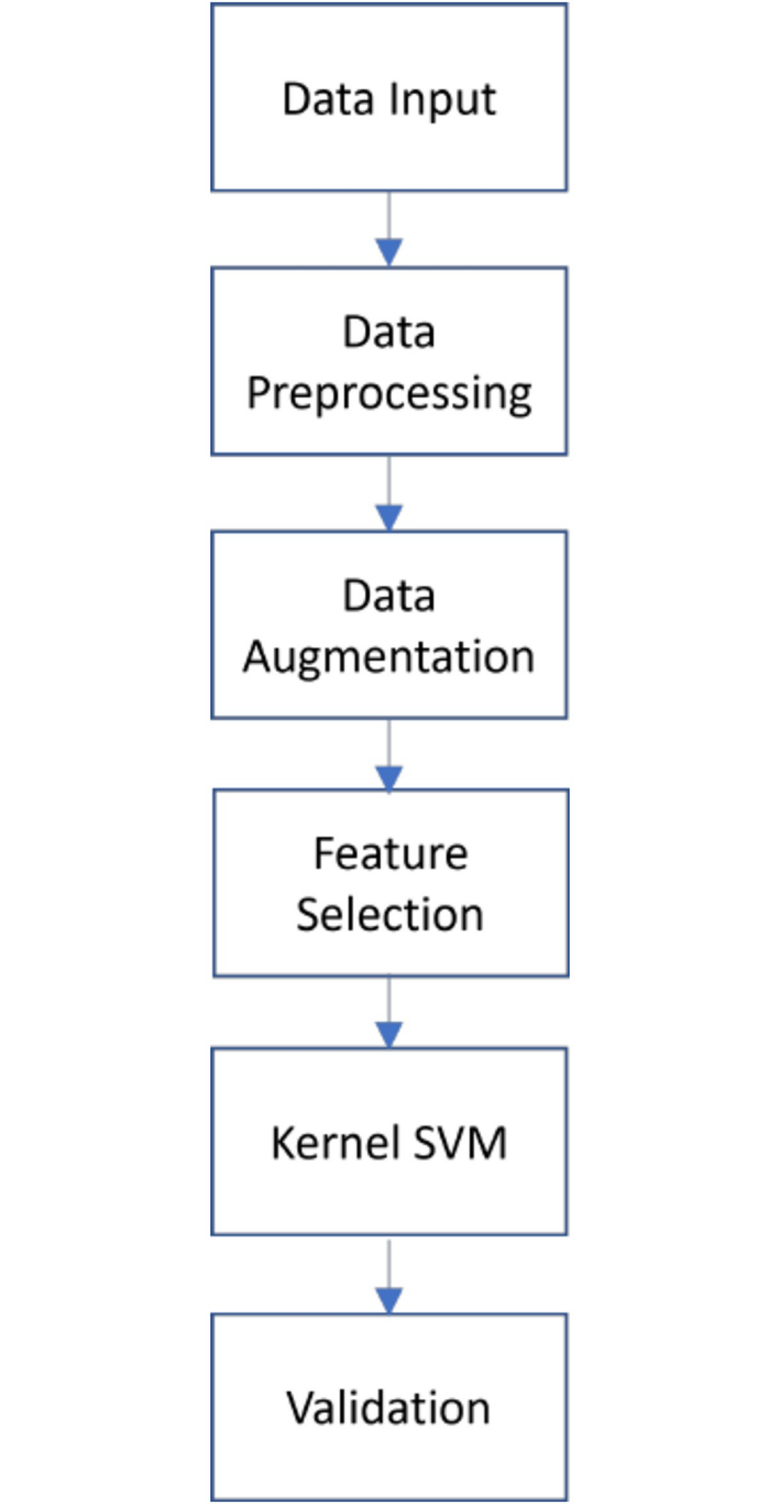
Conceptual design.

As illustrated in Fig 2, after preprocessing data, our work can be divided into three stages:

- Approach 1: The simplest models were built without any special technique application. These baselines models provide basic solution for further comparison with complex models applying different methods. In addition, four popular kernel types are applied to build models in this stage to define which kinds of kernel appropriate with the data. Kernel types that generate good results in prediction continue to be used for models in approaches 2 and 3.
- Approach 2: Baseline models are improved with the application of feature selection methods and hyperparameter tuning.
- Approach 3: The efficiency of balancing techniques is investigated by being applied to solve the problem of imbalanced data. After data is balanced, stages of approach 2 are repeated.

### Data collection and input

The dataset of Orange S.A Telcom Company is published on Kaggle. It contains historical records of 3333 customers in 51 state of the US, 19 features and one label (Table 2). The information of dataset is in papers [4, 6, 9, 15].

**Table 2:** Orange company dataset.

No.	Variables	Variable description	Variable types
1	State	51 states of the US	Categorical
2	Account length	Active duration of accounts	Integer
3	Area code	Code of areas	Categorical
4	International plan	1 = use service, 2 = not use service	Boolean
5	Voice mail plan	1 = use service, 2 = not use service	Boolean
6	Number vmail messages	Amount of voice mail messages	Integer
7	Total day minutes	Total day call minutes clients have used	Continuous
8	Total day calls	Total amount of day calls	Integer
9	Total day charge	Total day fee	Continuous
10	Total eve minutes	Total call minutes clients have used in the evening	Continuous
11	Total eve calls	Total amount of evening calls	Integer
12	Total eve charge	Total evening fee	Continuous
13	Total night minutes	Total night call minutes clients have used	Continuous
14	Total night calls	Total amount of night calls	Integer
15	Total night charge	Total night fee	Continuous
16	Total intl minutes	Total call minutes clients have used for international calls	Continuous
17	Total intl calls	Total amount of international calls	Integer
18	Total intl charge	Total International fee	Continuous
19	Customer service calls	Amount of customer service calls made	Integer
20	Churn	1 = churner, 0 = non-churner	Boolean

### Data preprocessing

#### Data visualization

**Fig 3:**
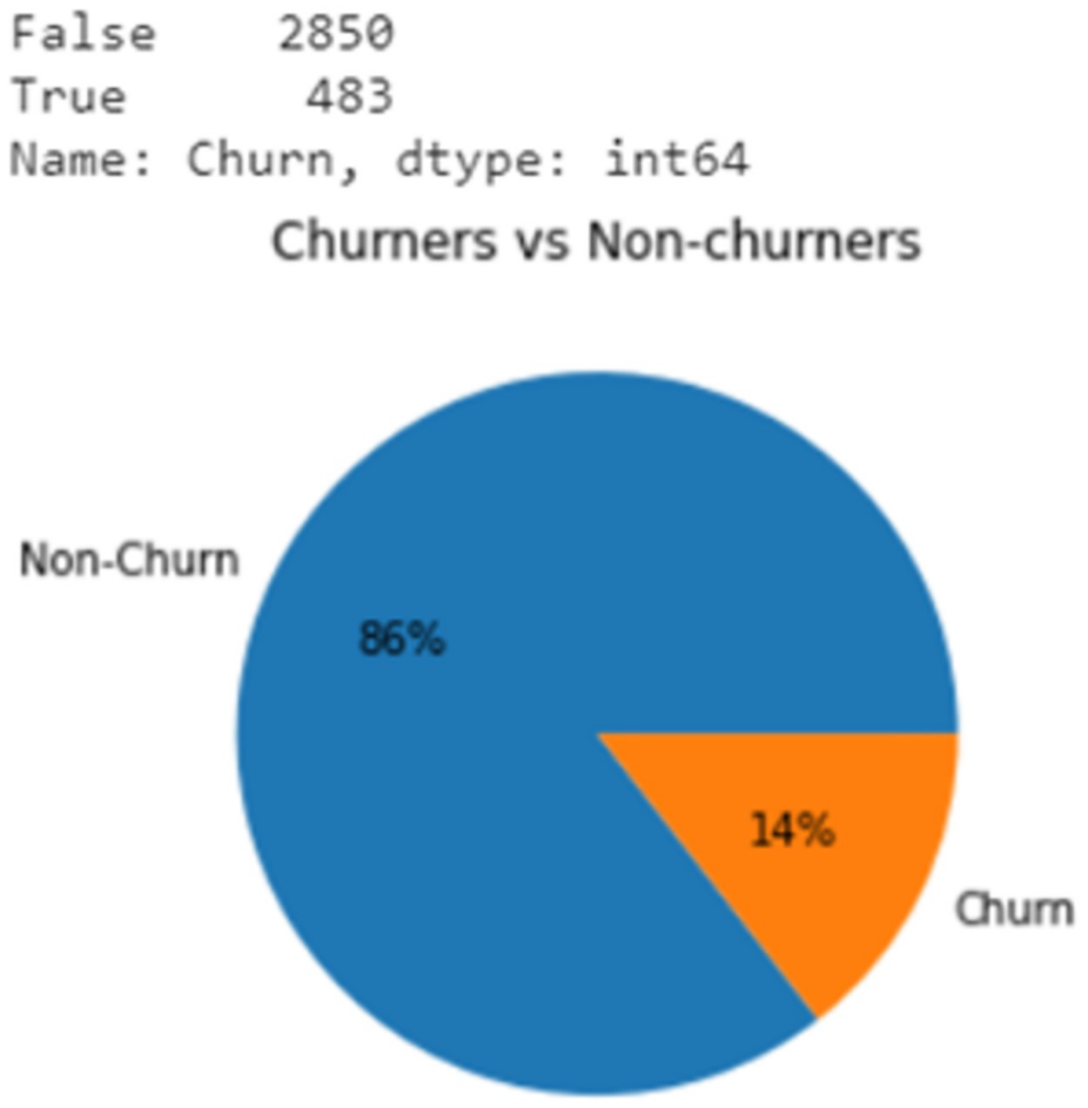
The ratio of churners to non-churners.

Data visualization is conducted to collect more information about the dataset. It can be seen clearly from Fig 3 that the dataset is highly imbalanced since the percentage of the group that represents churners is just account for 14% of the whole dataset. Non-churn group is dominant when its population is about 6 times more than that of Churn class. This causes errors in prediction and the bias for larger class because models are not provided enough data of smaller class to learn.

**Fig 4:**
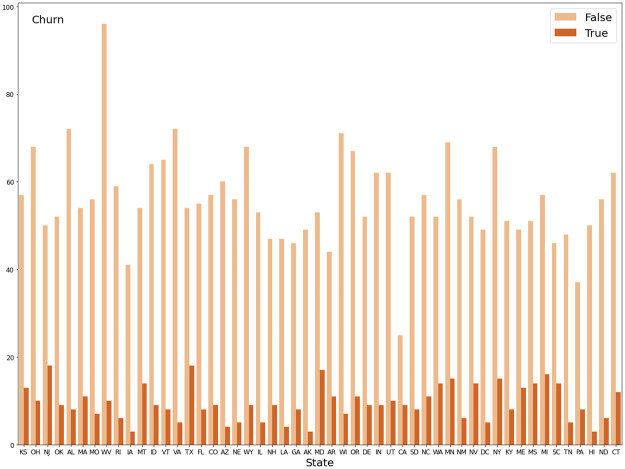
Count plot of target variable versus “State” variable.

**Fig 5:**
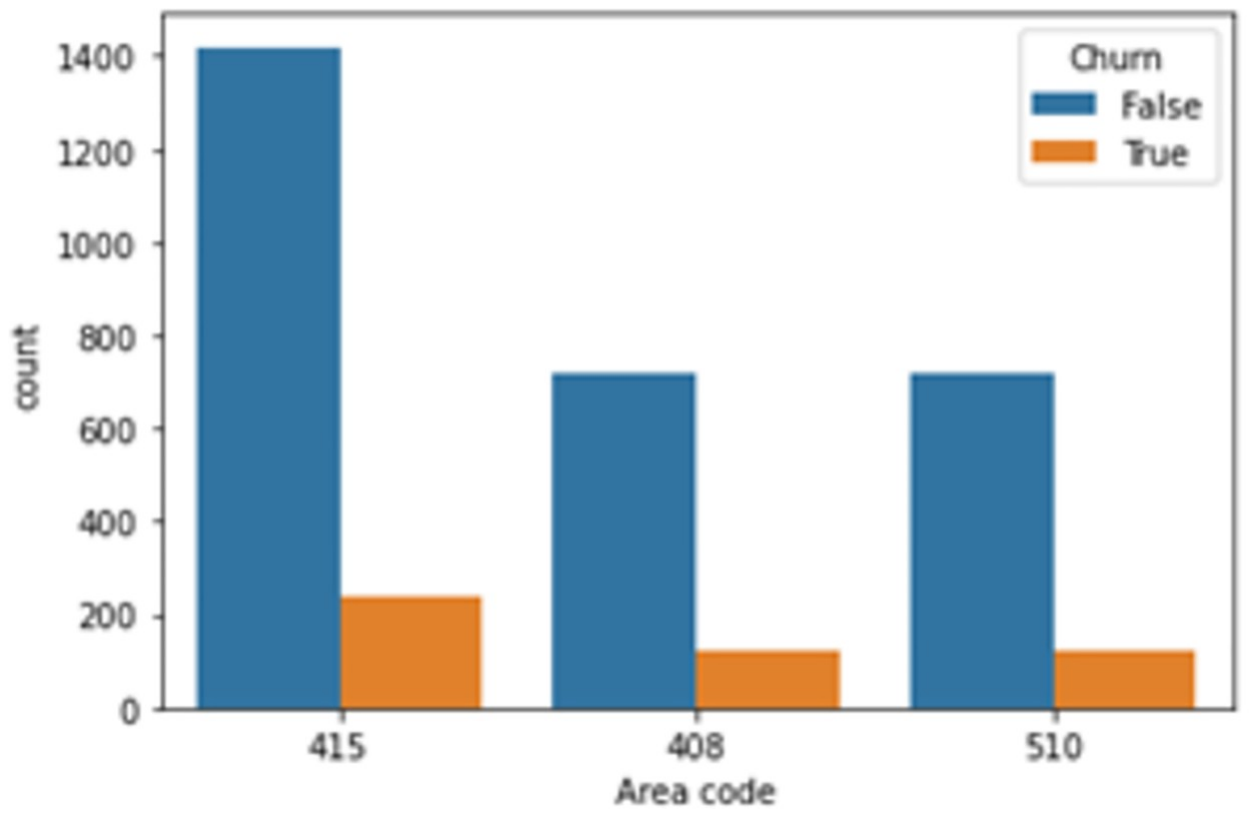
Count plot of target variable versus “Area code” variable.

On the other hand, Figs 4 and 5 show that two feature “State” and “Area code” provide mismatched information. There are three codes including 415, 408 and 510, which relate to San Francisco, San Jose, and the City of Oakland, respectively. However, this point conflicts with the information provided by the “State” variable since all three cities only belong to the State of California. Feature “State” illustrates that the data was collected across 51 states of the US. Data of “Area code” would be correct if the dataset only contained information of customers in California. Therefore, these two variables are dropped before doing further stages first because they do not provide any useful information and second is to avoid errors in the prediction.

#### Data standardization

It is important to standardize data as a pre-processing step to build a Machine Learning model, especially variables have varying scales. Z-score, which is calculated as formula below, is one of the most popular methods to standardize data. It makes data consistent by rescaling the values of variables be centered around the mean of 0 with standard deviation of 1.

z=x−μσ (1)

- x: the original score
- μ: the population mean
- σ: the population standard deviation.

### Feature selections

Feature Selection is taken to find out the optimal set of features for increasing the performance of models. In this study, Filter and Wrapper feature selection methods are applied to remove irrelevant, noisy, and redundant features.

#### Filter method

Filter methods evaluate the relationship between features and the label based on statistical calculation [17]. Pearson’s Correlation is one of the most commonly measurement to depict the dependence of two variables X and Y.


$$\rho_{X,Y}=\frac{\text{cov}\left(\text{X},\text{Y}\right)}{\sigma_{X}\sigma_{Y}}$$ (2)

It is noted that the more uncorrelated two features are, the closer to 0 correlation coefficient is. Otherwise, if a value is nearly to +1 and -1, two features have a strong positive and a strong negative correlation, respectively. Thus, two features are considered as highly related when their correlation coefficient is larger than 0.5 or smaller than -0.5; and one of them is dropped.

#### Wrapper method

In wrapper methods, subsets of features are alternately selected to train a model by applying Machine Learning algorithm and the results of prediction are evaluated to find out the optimal set of features. These methods are usually computationally expensive, but they give the more accuracy result than filter methods [17]. Sequential Forward Selection (SFS) and Sequential Backward Selection (SBF) are two popular wrapper methods for choosing features.

- Sequential Forward Selection (SFS): SFS algorithm is a bottom-up process starting with an empty set and then gradually adding the feature that generate the new most optimal feature set [18].
$$\text{J}\left({\text{X}}_{M}\right)=\text{max}\;\text{J}\left({\text{X}}_{M-1}\cup {\;\text{a}}_{i}\right),{\;\text{a}}_{i}\in S-{\text{X}}_{M-1}$$ (3)
  There are (M-l) features have been selected from the raw dataset S = {a_1_, a_2_,… a_n_} to form a new feature set X_*M*+1_. The next feature a_*i*_ is selected from the remaining features S − X_*M*+1_.
- Sequential Backward Selection (SBF): In contrast, SBS algorithm is a top-down process beginning with the raw feature set then removing sequentially the least significant feature that result in the smallest decline of the performance [18].
$$\text{J}\left({\text{X}}_{M}\right)=\text{max}\;\text{J}\left({\text{X}}_{M-1}-{\;\text{a}}_{i}\right),{\;\text{a}}_{i}\in {\text{X}}_{M+1}$$ (4)
  There are (n-M-1) features have been removed from the raw dataset S = {a_1_, a_2_,…a_n_} to form a new feature set X_*M*−1_. The next feature a_*i*_ is removed from feature set X_*M*−1_.

### Classification

#### Kernel Support Vector Machines

**Fig 6:**
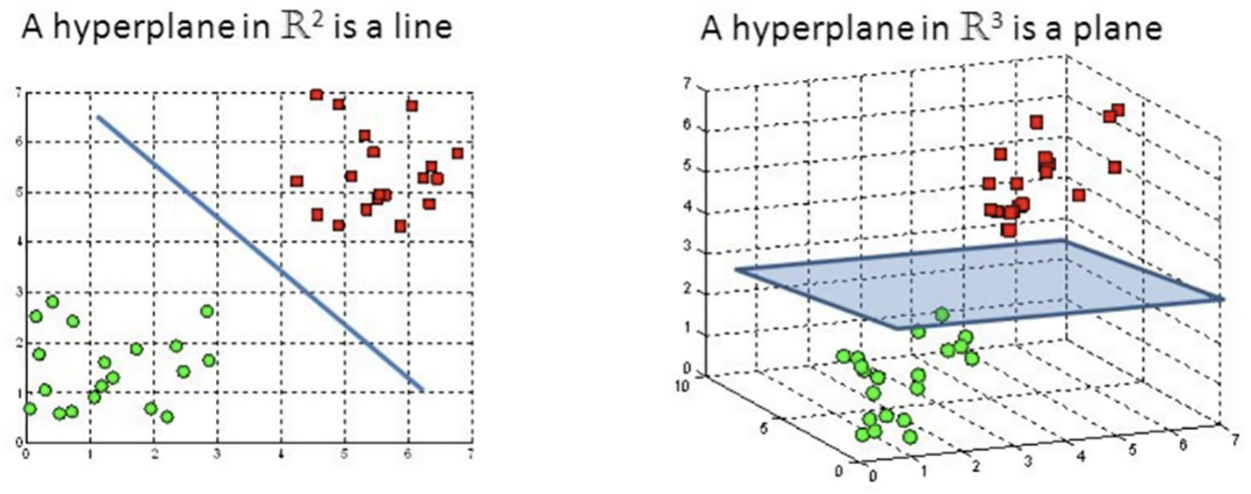
Hyperplanes.

Support Vector Machines (SVM) algorithm is extremely useful for classification of binary by identifying the correct hyperplanes (Fig 6), which is a boundary dividing space into two layers [19].

The hyperplanes:

$$\text{D}\left(\text{x}\right)={\text{w}}^{⊤}\text{x}+\text{b}\;\text{for}-1<\text{D}\left(\text{x}\right)<1$$ (5)

- w: is an m-dimensional vector of training set
- b: scalar and represents the bias
- ⊤: dot product operation

**Fig 7:**
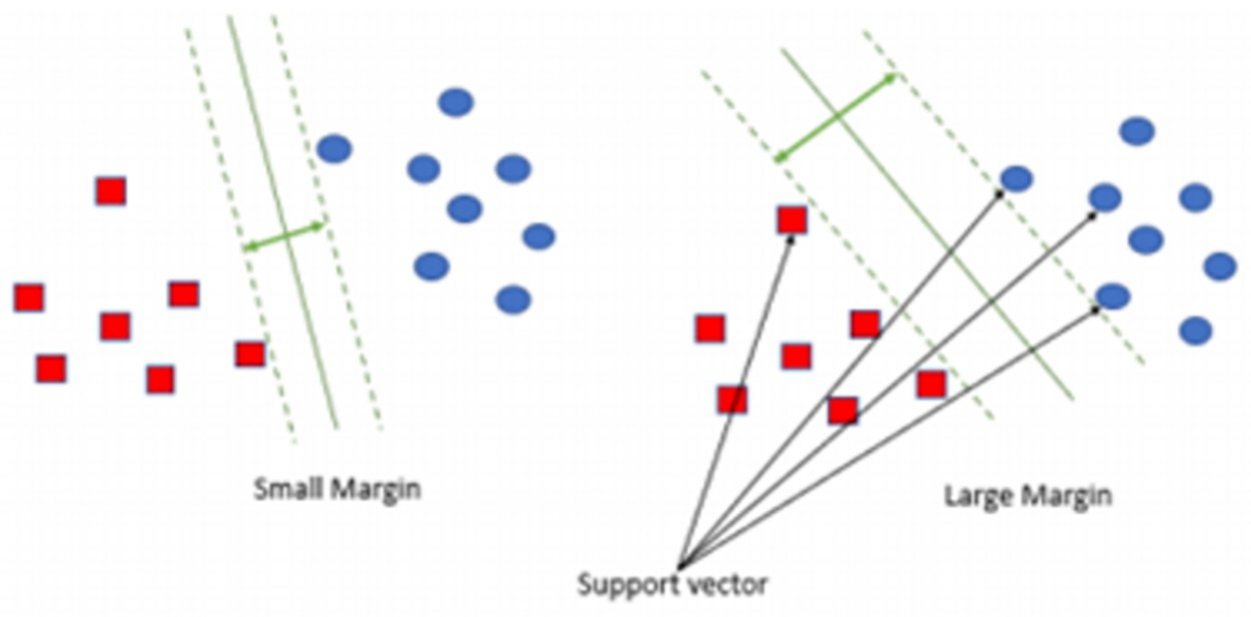
Margin and support vectors.

As illustrated in Fig 7, there are many hyperplanes on a surface and the data points which are closest to them are support vectors. Margin is also described in Fig 7 as the distance between two hyperplanes nearest data points from two different classes, including D(x) = 1 and D(x) = -1. It is noted that the margin is given by:

$$\frac{1}{\left|\left|w\right|\right|}+\frac{1}{\left|\left|w\right|\right|}=\frac{2}{\left|\left|w\right|\right|}$$ (6)

**Fig 8:**
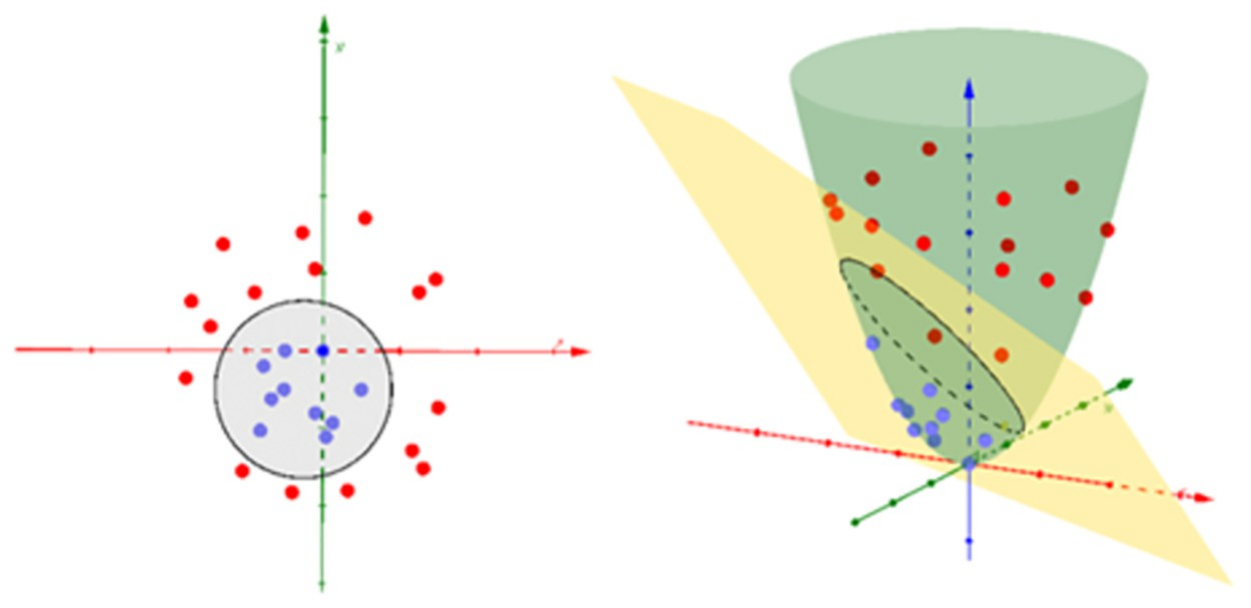
High-dimensional space transformation.

However, most real-world data are not linearly separated so it is impossible to identify the correct hyperplane in the input space. Therefore, Kernel Support Vector Machine (kernel SVM) is applied to transform the input space to a high-dimensional space (Fig 8). This method aims to find out a function **Φx** that transform data space of original input **x** a new feature space where data is separated linearly. The hyperplane formula in this case:

$$\text{D}\left(\text{x}\right)={\text{w}}^{⊤}\Phi \text{x}+\text{b}$$ (7)

Kernel function is formed into the equation below, here x and y are input vectors:

$$\text{K}\left(\text{x},\;\text{y}\right)=\Phi {\left(\text{x}\right)}^{⊤}\Phi \left(\text{y}\right)$$ (8)

There are four main popular kernel types in SVM algorithm.

- Linear kernel
$$\text{K}\;\left(\text{x},\;\text{y}\right)={\text{x}}^{⊤}\;\text{y}$$ (9)
  This simplest kernel form is used when the dataset is linearly separable. Thus, it is unnecessary to build a high-dimensional space. Linear kernel is the sum of the multiplication of each input pair of values.
- Polynomial (Poly) kernel
$$\text{K}\;\left(\text{x},\;\text{y}\right)={\left(\gamma {\;\text{x}}^{⊤}\text{y}+\text{r}\right)}^{\text{d}}\;\;\;\;\;\;\text{where}\;\gamma ,\;\text{d}>0$$ (10)
  This kernel type is more generalized than linear kernel due to the ability of separating the input space. The parameters include:
  - Gamma **γ**: There are two popular values for this parameter, one is $"\text{scale}"=\frac{1}{N*X.var())}$ which is also the default value. Another is $"\text{auto}"=\frac{1}{N}$, where N is number of features
  - Degree **d**: default value is 3. It is noted that this parameter is ignored by other kernel types.
  - The compromise coefficient **r**: This is the compromise between the impact of the more priority term against the term that is low-order. The default value = 0 denotes the homogeneity.
- Gaussian radial basis function (RBF) kernel
$$\text{K}\left(\text{x},\;\text{y}\right)=\text{exp}\left(-\gamma {\left\|\text{x}-\text{y}\right\|}^{2}\right),\;\;\text{Where}\;\gamma >0$$ (11)
  This is the most popular kernel and commonly used as RBF has the ability of mapping an input space in infinite dimensional space using Euclidean distance. It has one parameter that is Gamma **γ** to control the radius of the function. Similar to Poly kernel, it has two popular values that are “scale” and “auto”.
- Sigmoid kernel
$$\text{K}\;\left(\text{x},\;\text{y}\right)=\text{tanh}\left(\gamma {\;\text{x}}^{⊤}\text{y}+\text{r}\right)$$ (12)
  Sigmoid kernel is usually used in in neural networks for classification tasks as he hyperbolic tangent kernel or multilayer perceptron kernel. Its two parameters including Gamma **γ** and compromise coefficient **r** are the same as which of Poly kernel.

With the application of kernel tricks, SVM classifiers model is built with the objective of finding out the correct hyperplanes which maximizes the margin area to minimize the number of misclassifications.

Objective function:

w,b,ξmin12w2+C∑iNξi (13)

Subject to:

$${\text{y}}_{\text{i}}\left({\text{w}}^{⊤}\Phi \left({\text{x}}_{\text{i}}\right)+\text{b}\right)\geq 1-\xi_{\text{i}}\;\text{for}\;\text{i}=1\ldots \text{N},\Phi \left({\text{x}}_{\text{i}}\right)\;\text{where}\;\text{K}\left(\text{x},\;\text{y}\right)=\Phi {\left({\text{x}}_{\text{i}}\right)}^{⊤}\Phi \left({\text{y}}_{\text{i}}\right)$$ (14)

$$\xi_{\text{i}}\geq 0\;\text{for}\;\text{i}=1,2,3\ldots \text{N}$$ (15)

It is denoted that:

- ξ: slack variable
- For 0 < ξ ≤ 1: point is between margin and correct side of hyperplane. This is a margin violation.
- For ξ > 1: point is misclassified
- C: a regularization parameter, trade-off between two terms of objective function that are maximizing the margin and minimizing the number of misclassified variables.
- C value is small: large margin, the penalty for misclassification decreases.
- C value is large: narrow margin, the penalty for misclassification increases

### Hyperparameter tunning

The performance of a model depends highly on its hyperparameters, so it is important to define the values that generate the best result. Therefore, hyperparameter tuning is applied in our study to enhance the performance of models by searching over sets of values.

### Balancing techniques

In our study, balancing techniques are applied as the advanced approach to improve the performance of predictive models. Oversampling and under-sampling are two popular resampling methods for adjusting the population by adding or removing instances randomly to make classes in a dataset balanced [20]. Since these processes are conducted randomly without selection, under-sampling is possible to delete important data and keep unnecessary records while oversampling leads to overfitting due to copying exactly existing instances in the minority class. In addition, oversampling increased the smaller class until it equals the larger class. Thus, if the majority class contains many records, the dataset after applying oversampling will be enormous so it takes a lot of time to train models. Therefore, in this paper, we discuss different techniques that are combination of these two methods.

- Combination of Synthetic Minority Oversampling Technique and Tomek Link (SMOTE Tomek)

SMOTE Tomek is a hybridization between under-sampling and oversampling techniques [21]. Firstly, SMOTE is applied to increase the population of smaller classes based on K-Nearest Neighbor (KNN) algorithm. Instead of duplicating randomly existing minor data, new samples are generated between the selected point with its K-nearest points which are determined by calculating Euclidean Distance. However, overlap or overfitting may occur. Therefore, Tomek link is used to remove points of majority class that expand to the space of minority class with their nearest points of the minority for better class clusters. It is note that, contrast to basic under-sampling method, not only instances of majority class but also that of both classes are removed.

- Combination of Synthetic Minority Oversampling Technique and ENN (SMOTE ENN)

SMOTE ENN utilizes a similar concept as SMOTE TOMEK [21]. However, ENN determines points to be deleted based on KNN algorithm. In detail, if most K-nearest observations of selected point belonged to majority class are misclassified, the selected points will be removed from the dataset. SMOTE ENN may provide a cleaner dataset than SMOTE Tomek since there are more instances eliminated from the dataset.

### Computer software

We code our program using PYTHON language on a personal computer having a Core i7-7700, 8GB RAM, 128GB SSD.

### Result validation

The result validation based on the predictive result and actual labels is depicted by confusion matrices (Table 3) [15]:

**Table 3:** Confusion matrix.

		Prediction	
		Non-churn	Churn
Actual	Non-churn	TN True-Negative	FP False-Positive
	Churn	FN False-Negative	TP True-Positive

It is denoted that:

- True-positive (TP): points of positive class are exactly classified.
- False-positive (FP): points are classified into the positive class, but they actually belong to negative class
- True-negative (TN): points of negative class are exactly classified.
- False-negative (FN): points are classified into the negative class, but they actually belong to positive class

In our study, model performance is evaluated by two metric including Accuracy and F1 score. Accuracy is the ratio of correct classifications to total number of data points.

$$Accuracy=\frac{\text{TP}+\text{TN}}{TP+FP+FN+TN}$$ (16)

[16]

However, applying accuracy in an imbalanced dataset may lead to incorrect conclusion since it does not distinguish between the exact classified points of two classes and just simply takes the ratio of correct predictions over total observation. In this study, for evaluating models using unbalanced dataset, F1-score is better than Accuracy as it considers both false negative and false positive classifications. Below is the formular of F1-socre [17]:

$$Precision=\frac{\text{TP}}{TP+FP}$$ (17)

$$Recall=\frac{\text{TP}}{TP+FN}$$ (18)

$$F1\;\;score=\frac{2*\text{Precision}*\text{Recall}}{Precision+Recall}$$ (19)

- Precision: the ratio of true positive points over total points classified into positive class [16]
- Recall: the ratio of true positive points over total actual points of positive class [16]

## Results and discussion

The dataset in this study is divided into 80:20, which is 80% for training models and 20% for evaluating model performance. In approach 1, four types of kernel tricks are used in models and each case is tested five times, then the average performances are taken. From Table 4, Poly kernel and RBF kernel outperform in our studied case, so they continue to be used in further models.

**Table 4:** Average performance of baseline models.

	RBF	Linear	Poly	Sigmoid
Trial 1	0.6522	0	0.6269	0.1884
Trial 2	0.6757	0	0.6483	0.0946
Trial 3	0.5	0	0.5734	0.1159
Trial 4	0.6122	0	0.6928	0.1146
Trial 5	0.6143	0	0.6479	0.1111
Average F1 score	0.61088	0	0.63786	0.12492

Table 5 illustrates the results of feature selection stage. In approach 3, after resampling data by using SMOTE Tomek or SMOTE ENN, two algorithms SFS and SBS select the same feature subset. Filter feature selection method is not conducted in approach 3 since its efficiency is not high as wrapper feature selection methods

**Table 5:** The summary of feature selection results.

Features	Filter method	SFS	SFS	SFS/SBS (after SMOTE Tomek)	SFS/ SBS (after SMOTE ENN)
Account length	1	1	0	1	1
International plan	1	1	1	1	1
Voice mail plan	1	1	1	1	1
Number vmail messages	0	1	0	0	1
Total day minutes	0	1	1	1	1
Total day calls	1	0	0	1	1
Total day charge	1	1	1	1	1
Total eve minutes	0	1	1	1	1
Total eve calls	1	0	0	1	1
Total eve charge	1	1	0	1	1
Total night minutes	0	1	1	1	1
Total night calls	1	0	0	0	0
Total night charge	1	0	0	0	0
Total intl minutes	0	1	1	1	1
Total intl calls	1	1	1	1	1
Total intl charge	1	0	0	1	0
Customer service calls	1	1	1	1	1
Churn	labeled	labeled	Labeled	labeled	labeled
Total	12	12	9	14	14

1: feature is selected, otherwise 0.

Table 6 is the result summary of all models in this study. Several different techniques are investigated to increase the performance of churn prediction models using kernel SVM classifiers. The best hyperparameter values and metric scores of all models are recorded.

**Table 6:** The summary of model performances.

Techniques applied in SVM models	Parameters			Performance				
	C	γ	d	Accu -racy	Mis classification	Pre-cision	Recall	F1 score
RBF kernel baseline (ap1)	Df	Df	-	0.9370	0.0630	0.9444	0.9842	0.7529
Poly kernel baseline (ap1)	Df	Df	Df	0.9460	0.0540	0.9450	0.9947	0.7845
Filter + RBF kernel + Hyperparameter tuning (ap2)	4	scale	-	0.9400	0.0600	0.9522	0.9789	0.7752
Filter + Poly kernel + Hyperparameter tuning (ap2)	13	scale	3	0.9385	0.0615	0.9537	0.9754	0.7735
SFS + RBF kernel + Hyperparameter tuning (ap2)	5	scale	-	0.9460	0.0540	0.9556	0.9825	0.7978
SFS + Poly kernel + Hyperparameter tuning (ap2)	17	auto	3	0.9505	0.0495	0.9653	0.9772	0.8235
SBS + RBF kernel + Hyperparameter tuning (ap2)	7	scale	-	0.9490	0.0510	0.9605	0.9807	0.8132
SBS + Poly kernel + Hyperparameter tuning (ap2)	6	scale	5	0.9505	0.0495	0.9637	0.9789	0.8216
SMOTE Tomek + RBF kernel (ap3)	Df	Df	-	0.9359	0.0641	0.9191	0.9561	0.9345
SMOTE Tomek + Poly kernel (ap3)	Df	Df	Df	0.9263	0.0762	0.8953	0.9596	0.9236
SMOTE Tomek + SFS + RBF kernel + Hyperparameter tuning (ap3)	45	auto	-	0.9587	0.0413	0.9852	0.9316	0.9598
SMOTE Tomek + SBS+ RBF kernel + Hyperparameter tuning (ap3)								
SMOTE Tomek + SFS + Poly kernel + Hyperparameter tuning (ap3)	17	auto	4	0.9429	0.0571	0.9438	0.9421	0.9429
SMOTE Tomek + SBS + Poly kernel + Hyperparameter tuning (ap3)								
SMOTE ENN + RBF kernel (ap3)	Df	Df	-	0.9642	0.0358	0.9470	0.9716	0.9682
SMOTE ENN + Poly kernel (ap3)	Df	Df	Df	0.9520	0.0480	0.9178	0.9764	0.9567
SMOTE ENN + SFS + RBF kernel + Hyperparameter tuning (ap3)	60	scale	-	0.9888	0.0112	0.9976	0.9764	0.9901
SMOTE ENN + SBS+ RBF kernel + Hyperparameter tuning (ap3)								
SMOTE ENN + SFS + Poly kernel + Hyperparameter tuning (ap3)	21	auto	5	0.9714	0.0286	0.9805	0.9527	0.9751
SMOTE ENN + SBS + Poly kernel + Hyperparameter tuning (ap3)								

Df: default value, ap1: approach 1, ap2: approach 2, ap3: approach 3.

Initially, **in approach 1**, when kernel SVM models were built without any special technique applications and hyperparameters were kept at default values, RBF kernel SVM model achieved 0.75 F1 score and 0.94 accuracy score while Poly kernel SVM model achieved 0.78 and 0.95 for F1 score and accuracy score respectively. It is noted that before balancing techniques are conducted, F1-scores are more important than accuracy in evaluating models. Thus, the quality of baseline models is not good due to generating low F1 score and significant number of misclassifications. After that, feature selections were conducted to enhance the performance of models, **in approach 2**. The first strategy is to calculate Peason’s Correlation and redundant features were eliminated based on that coefficient. However, this approach seems not to be effective since it is difficult to identify the more important features in highly correlated feature pairs. This is reflected clearly in the result as Poly kernel SVM models are almost 1% less compared to its baseline model. In contrast, the results show that other algorithms for feature selections including SFS and SBF are active on the dataset. Models using feature sets selected from these methods and adjusted parameters achieved both higher F1-score and high accuracy. For this fundamental approach, Kernel Poly model with the application of parameter tuning and SFS or SBS gave the best result since it generated 0.82 F1 score, which is 4% and 7% higher than F1 score of Poly and RBF kernel SVM models respectively. The biggest challenge this study must deal with is the imbalance between the population of two customer groups in the given dataset. Therefore, **in approach 3**, resampling techniques, such as SMOTE Tomek and SMOTE ENN were applied as the improved solutions in this study. The outstanding improvement of the approach is reflected clearly from the results. After SMOTE Tomek and SMOTE ENN are applied, we use the same feature sets obtained from feature selection algorithms (SFS and SBS). From the result recorded, SMOTE ENN enhanced performance of models using the same other techniques better than SMOTE Tomek did. Especially, RBF kernel SVM model using parameter tuning (C = 60, Gamma = “scale”), balanced dataset generated by SMOTE ENN, and feature set chosen by SFS and SBS reached the best performance since it achieved an accuracy and F1 score of 99%. This model is better than models built in reference papers which use the same dataset with our study. As illustrated in Table 7, the highest accuracy achieved in paper [9] and [15] are 91.67% and 85.24% respectively.

**Table 7:** The results of reference papers.

Reference papers	Classifier	The best result
Zhou et al., 2019 [9]	Polynomial kernel Support Vector Machines	Accuracy: 91.67%
Jain et al., 2020 [15]	Logistics Regression	Accuracy: 85.24%, F1: 81.00%

**Fig 9:**
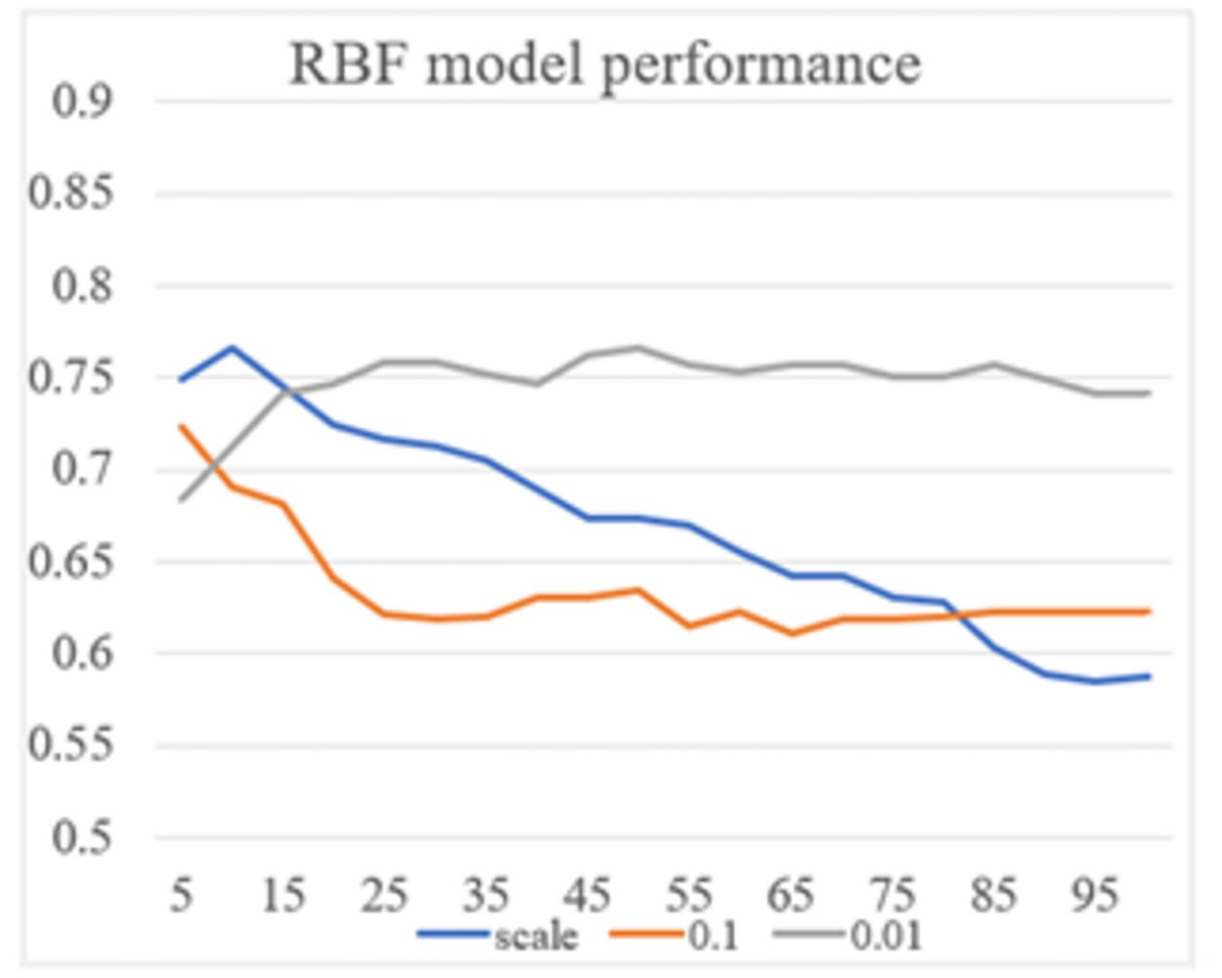
F1 scores of RBF kernel SVM model with different hyperparameter values.

Hyperparameter tunning plays an important role in building models using kernel Support Vector Machines. Each type of kernel has its own hyperparameters and these values affect significantly to the performance of models. This means that hyperparameters assigned different values would produce different results. An experiment is conducted to show how the performance of models varies due to the change of parameter values. A basic RBF kernel SVM model is built without any application of special techniques. Some values are tested for C and **γ**, which are two hyperparameters of RBF kernel. From the result illustrated in Fig 9, it can be seen clearly that the performance of the model fluctuates among the changes of hyperparameter values. With each pair of values [C, γ], the model generates a new F1 score. To minimize misclassifications, it is necessary to find out which values generate the best performance instead of using default values.

## Conclusion

This study aims to minimize the number of misclassifications in churn prediction by developing kernel Support Vector Machines models. Initial tests showed that RBF kernel and Poly kernel are appropriate to be used in this case. Several techniques including feature selection algorithms, resampling methods and hyperparameter tuning were gradually applied and combined to enhance the performance of models. Based on results obtained, RBF kernel SVM model with the applications of parameter tuning, SFS or SBS and SMOTE ENN outperforms other methods by achieving an accuracy of 99.01% and F1 score of 98.88%.

Despite having very high accuracy, our model is not optimal yet since some misclassification errors still exist. It is because Support Vector Machines highly depend on its hyperparameters. Our future research will be to expand the interval to search for better values of the hyperparameters. Furthermore, different machine learning algorithms can be applied in feature selections and resampling data.

## References

[1] Xu T, Ma Y, Kim K. Telecom Churn Prediction System Based on Ensemble Learning Using Feature Grouping. Applied Sciences. 2021;11(11):4742. doi: 10.3390/app11114742

[2] Mattison R. Churn Taxonomy. In: The telco churn management handbook. Oakwood Hills, IL: Xit Press; 2005. pp.33–61.

[3] Zhao Y, Li B, Li X, Liu W, Ren S. Customer Churn Prediction Using Improved One-Class Support Vector Machine. Advanced Data Mining and Applications. 2005;: 300–306. doi: 10.1007/11527503_36

[4] XIA G, JIN W. Model of Customer Churn Prediction on Support Vector Machine. Systems Engineering—Theory & Practice. 2008;28(1):71–77. doi: 10.1016/S18748651(09)60003-X

[5] Shaaban E., Helmy Y., Ayman E. Khder, Mona M. Nasr. A proposed Churn Prediction model. International Journal of Engineering Research and Applications (IJERA), 2012;2(4): 693–697. Available from: https://www.researchgate.net/publication/236625937_A_Proposed_Churn_Prediction_Model

[6] Brandusoiu I. and Toderean G. “Churn Prediction In The Telecommunications Sector Using Support Vector Machines,” in ANNALS OF THE ORADEA UNIVERSITY. Fascicle of Management and Technological Engineering, vol. XXII (XII), 2013/1, no. 1, 2013. doi: 10.15660/AUOFMTE.2013-1.2772

[7] Rodan Ali, Faris Hossam, Al-sakran Jamal, Al-Kadi Omar S. A Support Vector Machine Approach for Churn Prediction in Telecom Industry. International Interdisciplinary Journal, 2014:17(8). Available from: https://www.researchgate.net/publication/264534919_A_Support_Vector_Machine_Approach_for_Churn_Prediction_in_Telecom_Industry

[8] Keramati A, Jafari-Marandi R, Aliannejadi M, Ahmadian I, Mozaffari M, Abbasi U. Improved churn prediction in telecommunication industry using data mining techniques. Applied Soft Computing. 2014;24:994–1012. doi: 10.1016/j.asoc.2014.08.041

[9] Zhou L, Amoh DM., Boateng LK., Okine AA. Combined Appetency and Upselling Prediction Scheme in Telecommunication Sector Using Support Vector Machines. International Journal of Modern Education and Computer Science. 2019;11(6):1–7. doi: 10.5815/ijmecs.2019.06.01

[10] Ebrah K, Elnasir S. Churn Prediction Using Machine Learning and Recommendations Plans for Telecoms. Journal of Computer and Communications. 2019;07(11):33–53. doi: 10.4236/jcc.2019.711003

[11] Kriti. Customer churn: A study of factors affecting customer churn using machine learning. 2019. https://lib.dr.iastate.edu/creativecomponents/207

[12] Ullah I, Raza B, Malik A, Imran M, Islam S, Kim S. A Churn Prediction Model Using Random Forest: Analysis of Machine Learning Techniques for Churn Prediction and Factor Identification in Telecom Sector. IEEE Access. 2019;7:60134–60149. doi: 10.1109/ACCESS.2019.2914999

[13] Hu X, Yang Y, Chen L, Zhu S. Research on a Customer Churn Combination Prediction Model Based on Decision Tree and Neural Network. 2020 IEEE 5th International Conference on Cloud Computing and Big Data Analytics (ICCCBDA). 2020.

[14] Panjasuchat M, Limpiyakorn Y. Applying Reinforcement Learning for Customer Churn Prediction. Journal of Physics: Conference Series. 2020;1619:012016. doi: 10.1088/1742-6596/1619/1/012016

[15] Jain H, Khunteta A, Srivastava S. Churn Prediction in Telecommunication using Logistic Regression and Logit Boost. Procedia Computer Science. 2020;167:101–112. doi: 10.1016/j.procs.2020.03.187

[16] Panchal Mallika Naresh, Pandit Dr. Anala A. Churn Prediction using Supervised Machine Learning Algorithms—Impact of Oversampling. International Research Journal of Engineering and Technology (IRJET). 2020; 7(1). Available from: https://www.irjet.net/archives/V7/i11/IRJET-V7I11167.pdf

[17] Le M. T., Vo M. T., Pham N. T., and Dao S. V. Predicting heart failure using a wrapper-based feature selection. Indonesian Journal of Electrical Engineering and Computer Science. 2021; 21(3):1530–1539. doi: 10.11591/ijeecs.v21.i3.pp1530-1539

[18] Wei J, Xiao S, Dong W. Fault Location Method for Active Distribution Network Based on SVM and Feature Search Algorithm. 2019 IEEE 3rd International Electrical and Energy Conference (CIEEC). 2019.

[19] Mariette Awad, Rahul Khanna. Support Vector Machines for Classification. In: Efficient Learning Machines. Apress, Berkeley, CA, 2015. pp 39–66.

[20] Sasada T, Liu Z, Baba T, Hatano K, Kimura Y. A Resampling Method for Imbalanced Datasets Considering Noise and Overlap. Procedia Computer Science. 2020;176:420–429. doi: 10.1016/j.procs.2020.08.043

[21] Batista G, Prati R, Monard M. A study of the behavior of several methods for balancing machine learning training data. ACM SIGKDD Explorations Newsletter. 2004;6(1):20–29. doi: 10.1145/1007730.1007735

